# Supplementary material for: The evolution of competitive ability for essential resources
Source: Philos Trans R Soc Lond B Biol Sci. 2020 Mar 23;375(1798):20190247. doi: 10.1098/rstb.2019.0247 (PMC7133530; doi:10.1098/rstb.2019.0247)
Supplement: Appendix C: Supplementary Tables [file rstb20190247supp3.pdf]

Electronic Supplementary Materials: Appendix C

Supplementary Tables

Tables S1-S7

Evolution of competitive ability for essential resources

Joey R. Bernhardt[1*], Pavel Kratina[2], Aaron L. Pereira[1], Manu Tamminen[3], Mridul K. Thomas[4], Anita Narwani[1]

[1]Aquatic Ecology Department, Eawag, Überlandstrasse 133, CH-8600 Dübendorf, Switzerland

[2]School of Biological and Chemical Sciences, Queen Mary University of London, Mile End Road, London E1 4NS, United Kingdom.

[3]Department of Biology, University of Turku, Natura, University Hill, 20014 Turku, Finland

[4]Centre for Ocean Life, DTU Aqua, Technical University of Denmark, Kongens Lyngby, Denmark

*Correspondence to: joey.bernhardt@biodiversity.ubc.ca

	Change in I^*		
	(1)	(2)	(3)
**Change in salt tol**	-0.21 (-0.57, 0.15)		
**Change in *N****		0.38^{**} (0.07, 0.68)	
**Change in *P****			0.34^{*} (-0.03, 0.72)
**Change in size**	0.11 (-0.25, 0.47)	0.04 (-0.30, 0.37)	0.10 (-0.24, 0.44)
**Anc 3**	-0.59 (-1.60, 0.41)	-0.66 (-1.58, 0.27)	-0.44 (-1.43, 0.55)
**Anc 4**	-0.35 (-1.30, 0.60)	0.11 (-0.84, 1.07)	-0.29 (-1.20, 0.63)
**Anc 5**	1.23^{**} (0.09, 2.36)	1.35^{**} (0.31, 2.39)	1.30^{**} (0.21, 2.39)
**cc1690**	-0.77 (-1.81, 0.27)	-0.64 (-1.59, 0.31)	-0.75 (-1.75, 0.25)
**Constant**	0.07 (-0.69, 0.83)	-0.21 (-0.88, 0.47)	0.11 (-0.61, 0.83)
Observations	32	32	32
R^2	0.46	0.54	0.49
Adjusted R^2	0.33	0.43	0.37
Note:			$^{*}p<0.1$; $^{**}p < 0.05$; $^{***}p<0.01$; (95% CI)

Table S1. Multiple regression fits of change in I^* of descendant populations relative to their ancestors, as a function of changes in cell biovolume (size; when growing in light limiting conditions (ESM Figure S9) and ancestry, as well as changes in salt tolerance (model 1), changes in N^* (model 2), changes in P^* (model 3).

	Change in P^*		
	(1)	(2)	(3)
**Change in salt tol**	-0.13 (-0.49, 0.24)		
**Change in I^***		0.38^* (0.001, 0.75)	
**Change in N^***			0.13 (-0.20, 0.45)
**Change in size**	0.05 (-0.33, 0.43)	-0.08 (-0.45, 0.29)	0.01 (-0.37, 0.39)
**Anc 3**	-0.58 (-1.60, 0.44)	-0.41 (-1.39, 0.57)	-0.63 (-1.64, 0.39)
**Anc 4**	-0.18 (-1.17, 0.81)	-0.12 (-1.05, 0.81)	-0.05 (-1.10, 1.01)
**Anc 5**	-0.23 (-1.45, 1.00)	-0.87 (-2.14, 0.41)	-0.23 (-1.44, 0.99)
**cc1690**	-0.05 (-1.01, 0.92)	0.32 (-0.65, 1.28)	0.04 (-0.94, 1.01)
**Constant**	-0.39 (-1.14, 0.36)	-0.40 (-1.09, 0.28)	-0.51 (-1.25, 0.23)
Observations	32	32	32
R^2	0.10	0.20	0.10
Adjusted R^2	-0.12	0.01	-0.12
Note:			$^*p<0.1$; $^{**}p < 0.05$; $^{***}p<0.01$; (95% CI)

Table S2. Multiple regression fits of change in P^* of descendant populations relative to their ancestors, as a function of changes in cell biovolume (size; when growing in phosphorus limited conditions (ESM Figure S7), ancestry and changes in salt tolerance (model 1), changes in I^* (model 2), changes in N^* (model 3).

	Change in N^*		
	(1)	(2)	(3)
**Change in salt tol**	-0.15 (-0.60, 0.30)		
**Change in *I****		0.56^{**} (0.14, 0.97)	
**Change in *P****			0.20 (-0.28, 0.68)
**Change in size**	0.004 (-0.45, 0.46)	-0.09 (-0.48, 0.31)	-0.08 (-0.52, 0.37)
**Anc 3**	-0.06 (-1.28, 1.15)	0.30 (-0.82, 1.42)	0.03 (-1.21, 1.27)
**Anc 4**	-1.28^{**} (-2.50, -0.06)	-1.15^{**} (-2.23, -0.06)	-1.31^{**} (-2.51, -0.10)
**Anc 5**	-0.68 (-1.90, 0.53)	-1.25^{**} (-2.41, -0.08)	-0.61 (-1.83, 0.62)
**cc1690**	-0.53 (-1.70, 0.63)	-0.02 (-1.12, 1.09)	-0.50 (-1.66, 0.66)
**Constant**	0.61 (-0.32, 1.54)	0.48 (-0.31, 1.26)	0.57 (-0.31, 1.46)
Observations	32	32	32
R^2	0.21	0.37	0.22
Adjusted R^2	0.02	0.22	0.03
Note:			$^{*}p<0.1$; $^{**}p < 0.05$; $^{***}p<0.01$; (95% CI)

Table S3. Multiple regression fits of change in N^* of descendant populations relative to their ancestors, as a function of changes in cell biovolume (size; when growing in nitrogen limited conditions (ESM Figure S8), ancestry, and changes in salt tolerance (model 1), changes in I^* (model 2), changes in P^* (model 3).

	Competitive ability for nitrogen, CN (1/*N**)
**CP**	0.36** (0.02, 0.70)
**CI**	0.08 (-0.26, 0.42)
**Biovolume**	0.02 (-0.32, 0.36)
$\boldsymbol{\mu_{max}}$	-0.37* (-0.74, 0.01)
**Anc 3**	-0.10 (-1.12, 0.92)
**Anc 4**	-0.73 (-1.68, 0.22)
**Anc 5**	-0.30 (-1.29, 0.70)
**cc1690**	-0.20 (-1.17, 0.77)
**Constant**	0.28 (-0.42, 0.97)
Observations	37
R^2	0.37
Adjusted R^2	0.18
Note:	*$p<0.1$; **$p < 0.05$; ***$p<0.01$; (95% CI)

Table S4. Multiple regression fits of competitive ability for nitrogen (CN), as a function of competitive ability for phosphorus (CP), competitive ability for light (CI), cell biovolume when growing in nitrogen limited conditions (ESM Figure S8) and u_{max} (derived from fits of Monod curve over a gradient of nitrogen supply; Figure 2B).

	Competitive ability for light, CI (1/*I**)
**CP**	-0.24 (-0.64, 0.16)
**CN**	-0.14 (-0.53, 0.26)
**Biovolume**	-0.50** (-0.87, -0.12)
μ_{max}	-0.15 (-0.52, 0.22)
**Anc 3**	-0.08 (-1.27, 1.11)
**Anc 4**	0.32 (-0.83, 1.47)
**Anc 5**	-0.01 (-1.30, 1.27)
**cc1690**	-0.33 (-1.47, 0.82)
**Constant**	0.12 (-0.74, 0.99)
Observations	32
R^2	0.34
Adjusted R^2	0.11
Note:	$^{*}p$<0.1; $^{**}p$ < 0.05; $^{***}p$<0.01; (95% CI)

Table S5. Multiple regression fits of competitive ability for light (CI), as a function of competitive ability for nitrogen (CN), competitive ability for light (CI), cell biovolume when growing in light limited conditions (ESM Figure S9) and umax (derived from fits of Monod curve over a gradient of light availability; Figure 2C).

	Competitive ability for phosphorus, CP (1/*P**)
**CN**	0.54*** (0.24, 0.84)
**CI**	0.07 (-0.25, 0.38)
**Biovolume**	0.28 (-0.05, 0.61)
μ_{max}	-0.35** (-0.64, -0.07)
**Anc 3**	-0.28 (-1.17, 0.60)
**Anc 4**	0.46 (-0.43, 1.35)
**Anc 5**	-0.23 (-1.13, 0.67)
**cc1690**	-0.32 (-1.21, 0.56)
**Constant**	0.07 (-0.57, 0.70)
Observations	37
R^2	0.48
Adjusted R^2	0.33
Note:	*$p<0.1$; **$p < 0.05$; ***$p<0.01$; (95% CI)

Table S6. Multiple regression fits of competitive ability for phosphorus (CP), as a function of competitive ability for nitrogen (CN), competitive ability for light (CI), cell biovolume when growing in phosphorus limited conditions (ESM Figure S7) and u_{max} (derived from fits of Monod curve over a gradient of phosphorus supply; Figure 2A).

	B	BS	C	L	N	P	S
Anc2	484	473	NA	473	476	475	436
Anc3	450	415	NA	396	419	487	433
Anc4	530	500	468	492	513	530	508
Anc5	563	NA	582	533	537	563	569

Table S7. Number of variable SNPs between the ancestors and descendants from different selection environments: C: COMBO, L: light-limited, P: P-limited, N: N-limited, B: biotically depleted media, S: high salt, BS: biotically depleted and high salt.
